# Fast Focal Point Correction in Prism-Coupled Total Internal Reflection Scanning Imager Using an Electronically Tunable Lens

**DOI:** 10.3390/s18020524

**Published:** 2018-02-09

**Authors:** Chenggang Zhu, Bilin Ge, Ru Chen, Xiangdong Zhu, Lan Mi, Jiong Ma, Xu Wang, Fengyun Zheng, Yiyan Fei

**Affiliations:** 1 Department of Optical Science and Engineering, Shanghai Engineering Research Center of Ultra-Precision Optical Manufacturing, Key Laboratory of Micro and Nano Photonic Structures (Ministry of Education), Fudan University, Shanghai 200433, China; 16110720026@fudan.edu.cn (C.Z.); 17210720001@fudan.edu.cn (B.G.); 15210720009@fudan.edu.cn (R.C.); lanmi@fudan.edu.cn (L.M.); jiongma@fudan.edu.cn (J.M.); 2 Department of Physics, University of California, Davis, CA 95616, USA; xdzhu@physics.ucdavis.edu; 3 Department of Fundamental Courses, Wuxi Institute of Technology, Wuxi 214121, China; wangxu@wxit.edu.cn; 4 Institutes of Biomedical Science, Fudan University, Shanghai 200032, China; zhengfengyun@outlook.com

**Keywords:** total internal reflection, focal point correction, electronically tunable lens, protein microarray, label-free optical biosensors

## Abstract

Total internal reflection (TIR) is useful for interrogating physical and chemical processes that occur at the interface between two transparent media. Yet prism-coupled TIR imaging microscopes suffer from limited sensing areas due to the fact that the interface (the object plane) is not perpendicular to the optical axis of the microscope. In this paper, we show that an electrically tunable lens can be used to rapidly and reproducibly correct the focal length of an oblique-incidence scanning microscope (OI-RD) in a prism-coupled TIR geometry. We demonstrate the performance of such a correction by acquiring an image of a protein microarray over a scan area of 4 cm^2^ with an effective resolution of less than 20 microns. The electronic focal length tuning eliminates the mechanical movement of the illumination lens in the scanning microscope and in turn the noise and background drift associated with the motion.

## 1. Introduction

Total internal reflection (TIR) is useful for interrogating physical and chemical processes at the interface between two transparent media. When an illumination light propagating through a medium of a refractive index n_1_ meets an interface with a second medium of a refractive index n_2_ < n_1_, TIR occurs for angles of incidence θ greater than the critical angle θ_c_ = arcsin(n_2_/n_1_). Despite being totally reflected, the illumination light establishes an evanescent electromagnetic field that penetrates into the second medium and decays exponentially with the distance from the interface [1,2]. The penetration depth of the evanescent wave is on the order of the incident wavelength, which restricts the illuminating light to an extremely thin region in the second medium. TIR-based technologies use the evanescent wave to interact with samples within the thin region so that TIR provides an enhanced surface sensitivity [3]. Various TIR geometry-based spectroscopies and imaging techniques, including IR and UV/vis adsorption, Raman scattering, and fluorescence, have found extensive applications in examining cell-substrate contacts, protein dynamics, and biomolecular interactions [4,5,6,7,8].

In a typical TIR-based surface sensitive detection, a prism is routinely used to produce an evanescent wave and afford convenient control of the incidence angle and field of view [1,3,9]. This makes the TIR-based surface sensitive imaging techniques suitable for large area detection [7,9,10,11,12]. For the latter, one can use a collimated beam to illuminate an area of interest and image the reflection beam onto a CCD (Charge-coupled Device) camera [13]. Due to image distortion and the loss of spatial resolution at oblique-incidence, the sensing area is typically limited to ~1 cm^2^ [14,15]. To acquire large images at oblique-incidence, one can instead scan a focused beam over the area of interest and detect the reflected beam from one pixel at a time [8,16]. The challenge of such a scanning microscope is to keep the focus of the illumination beam on the surface when the distance the beam travels inside the prism before incidence on the surface varies. To meet this challenge, Landry et al., Malovichko et al., and Andrew et al. chose to move the illumination lens with an encoded linear stage to maintain the focal point on the surface [16,17,18,19]. The drawbacks of this compensation method are the cost, the bulkiness of the illumination optics, and the extra noise introduced by the mechanical motion of the illumination lens. It is desirable to do away with mechanically moving the illumination lens while still be able to maintain the focus of the beam on the surface.

In this paper, we show that an electrically tunable lens can be employed to move the focal point of the illumination beam without moving the illumination lens. By imaging a protein microarray over an area of 4 cm^2^ with an oblique-incidence scanning microscope configured in the TIR geometry, we illustrate that the image blurring can indeed be corrected efficiently with a combination of an electronically tunable lens and the f-theta scan lens as the composite illumination lens. Furthermore, by acquiring real-time binding curves that require repeatedly and rapidly adjusting the electronically tunable lens, we demonstrate that such a compensation scheme indeed provides reproducible focal correction and eliminates the noise and drift in the optical signal associated with the mechanical motion of the illumination lens.

## 2. Materials and Methods

### 2.1. Total Internal Reflection Oblique-Incidence Reflectivity Difference Microscope (TIR OI-RD)

**Figure 1:**
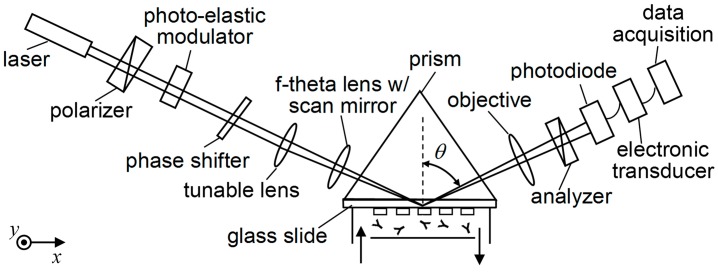
Schematic diagram showing total internal reflection oblique-incidence reflectivity difference (TIR OI-RD) for the label-free detection of a microarray. A prism enables the illumination beam to undergo total internal reflection and focus on the backside of the glass slide. With a combination of beam scanning along the y-axis and sample movement along the x-axis, the TIR OI-RD microscope is able to investigate tens of thousands of biomolecular reactions in a single experiment. An electronically tunable lens is used for focal point correction to obtain a good quality, large microarray image.

An oblique-incidence reflectivity difference (OI-RD) scanning microscope is an ellipsometry-based biosensor. It is capable of detecting a biomolecular microarray with tens of thousands of features on a solid support [20,21,22,23,24]. To take advantage of an electrically tunable lens, we develop a prism-coupled OI-RD scanning microscope, as shown in Figure 1. The microscope characterizes the thickness, d, of a molecular layer through the change in phase difference between the *p-* and *s*-polarized components of the reflected light beam caused by such a layer [20,22]:

(1) $$\Delta {\delta =\delta -\delta }_{0}≅\frac{-4\pi \sqrt{\varepsilon_{s}}\cos\theta }{\left(\varepsilon_{0}-\varepsilon_{s}\right)\left(\cot^{2}{\theta -\varepsilon }_{s}/\varepsilon_{0}\right)}\frac{\left(\varepsilon_{d}-\varepsilon_{0}\right)\left(\varepsilon_{d}{-\varepsilon }_{s}\right)}{\varepsilon_{d}}\frac{d}{\lambda }$$

$\delta_{0}$ and δ represent the phase difference between the *p-* and *s*-polarization from the bare solid surface and from the solid surface covered with the molecular layer, respectively. The wavelength λ is 632.8 nm for the He-Ne laser used in this microscope. $\varepsilon_{s}$, $\varepsilon_{0}$, and $\varepsilon_{d}$ are optical dielectric constants of the buffer liquid, solid support or substrate, and molecular layer. The substrate is made of BK7 glass with $\varepsilon_{0}=2.30$. The buffer is an aqueous phosphate-buffered saline (PBS, Sigma-Aldrich, Shanghai, China) with $\varepsilon_{s}$ = 1.77 at 632.8 nm. The incident angle θ is 63.3°, exceeding the critical angle θ_c_ = 61.4° for the interface of BK7 glass and the PBS buffer. As illustrated in Figure 1, we place a BK7 equilateral prism (Gousoptics, Shanghai, China) on top of a glass slide. A layer of index-matching fluid with an optical dielectric constant of 2.31 at 632.8 nm (Cargille Labs, Cedar Grove, NJ, USA) is added between the prism and the glass slide. We fabricate the microarray of biomolecules on the opposite side of the glass slide in contact with the PBS buffer solution. The width of the prism (along the *x*-axis) is 18 mm and the edge of the prism (along the *y*-axis) is 52 mm. The illumination beam enters one side of the prism and is focused on the microarray-bearing surface. The reflected beam exits the prism and is imaged onto a photodiode with an objective lens. We acquire an image of a bimolecular microarray by scanning the focused beam across the microarray along the *y*-axis (the vertical direction) with a combination of an f-theta lens and a galvo-mirror, as well as by moving the microarray with an encoded translation stage along the *x*-axis (the horizontal direction). To maintain the focus of the illumination beam on the microarray surface as the translation stage moves along the *x*-axis, we add an electronically tunable lens (EI-10-30-CI-VIS-LD, Optotune, Dietikon, Switzerland) 170 mm in front of the f-theta lens. The tunable lens is a shape-changing polymer lens whose curvature is adjustable by applying an electric current. The tunable lens offers a continuous adjustment of focal powers from −1.5 dpt to 3.5 dpt and in turn yields a focal length from −660 mm to infinity and from infinity to +285 mm. It has a resolution of 0.002 dpt at a response time of 10 ms.

### 2.2. Microarray Fabrication and Reaction

A microarray of 5936 (53 × 112) biotin-conjugated bovine serum albumin (BBSA, Sigma-Aldrich) is printed on an epoxy-functionalized glass slide (CapitalBio Corporation, Beijing, China) at a concentration of 7.6 μM in 1 × PBS. The spot diameter is around 150 μm and the center-to-center spacing between two adjacent spots is 250 μm. The microarray covers an area of 13 mm × 28 mm. We install a microarray slide in a fluidic assembly and image it with the TIR OI-RD scanning microscope. The printed microarray is washed in situ with 1 × PBS buffer to remove excess printed materials and is incubated in a solution of 7.6 μM bovine serum albumin (BSA, Sigma-Aldrich, Shanghai, China) for 30 min to cover the unprinted surface. We react mouse anti-biotin IgG (Jackson ImmunoResearch, West Grove, PA, USA) with the microarray as follows. With the microarray initially in 1 × PBS buffer, we replace the buffer with a solution of anti-biotin antibody at a flow rate of 5 mL/min for 18 s. Afterward, the flow rate is reduced to 0.05 mL/min and continues for 20 min (association phase of the reaction). We then replace the antibody solution with 1 × PBS buffer at the rate of 5 mL/min in 18 s, after which we reduce the flow rate to 0.05 mL/min and continue the flow for another 40 min (dissociation phase of the reaction).

### 2.3. Microarray Detection with the TIR OI-RD Scanning Microscope

We acquire OI-RD images of the microarray before and after the reaction for endpoint analysis. With a step size of 18.7 µm along two orthogonal directions, there are 710 pixels along the *x*-axis and 1500 pixels along the *y*-axis in an endpoint image of the large microarray with 5936 BBSA spots. It is obtained by (1) moving the illumination beam 1500 steps along the *y*-axis, with each step moving 18.7 µm and acquiring OI-RD signals; (2) returning the illumination beam to the starting position on the *y*-axis; (3) adjusting the focal power of the tunable lens with a pre-determined value; (4) moving the microarray surface (on the translation stage) forward by one step at the step size of 18.7 µm along the *x*-axis; (5) moving the illumination beam 1500 steps along the *y*-axis as in (1); and (6) repeating (3) through (5) until all 710 positions along the *x*-axis have been interrogated. With the aforementioned scanning procedure, it takes about 20 min to acquire an OI-RD image with 1 ms spent on each movement of the illumination beam and data acquisition and 100 ms spent on each movement of the translation stage.

To measure the association-dissociation curves on all 5936 immobilized BBSA targets, we select one pixel from each target spot as the signal channel and two pixels in the neighboring unprinted region along the y-axis as the reference channels. The optical signal Δδ from a signal channel minus the averaged signal from the two neighboring reference channels yields the background-corrected signal. All curves (on target spots and on reference regions) are obtained by (1) moving the illumination beam 225 steps along the y-axis, with each step moving 125 µm and acquiring OI-RD signals, including 112 signals from targets and 113 signals from substrate references; (2) returning the illumination beam to the starting position on the y-axis; (3) adjusting the focal power of the focus tunable lens with a pre-determined value; (4) moving the translation stage forward by one step at the step size of 250 µm along the x-axis in order to read signals from target spots 250 µm away from the previous reading; (5) moving the illumination beam 225 steps along the y-axis as in (1); (6) repeating (3) through (5) until all 53 positions along the x-axis have been interrogated; and (7) repeating 94 iterations from (1) through (6) with 11,925 pixels (53 × 225) in one iteration.

## 3. Results

### 3.1. Protein Microarray Image Acquired with the Prism-Coupled TIR OI-RD Scanning Microscope without Focal Correction

**Figure 2:**
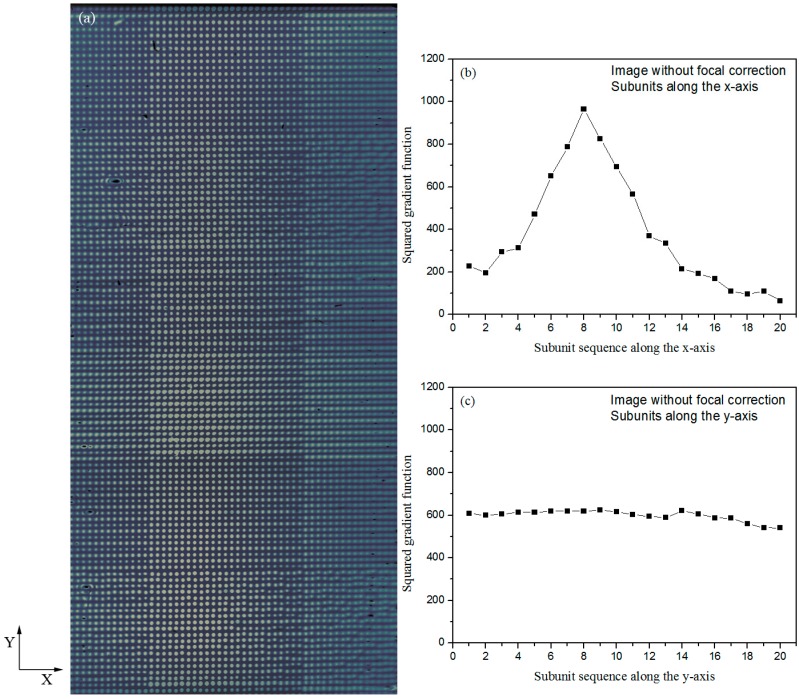
(**a**) A BBSA (biotin-conjugated bovine serum albumin) microarray image obtained with the TIR OI-RD microscope without focal correction. Image sharpness changes gradually along the x-axis; (**b**) Squared gradient functions of 20 subunits along the *x*-axis, each having 35 × 1500 pixels. The subunit sequence begins from the left side of the microarray image. The eighth subunit has the largest squared gradient function, indicating the best focusing in this region; (**c**) Squared gradient functions of 20 subunits along the *y*-axis, each having 710 × 75 pixels. The subunit sequence begins from the top of the microarray image.

Figure 2a shows the image of a BBSA microarray without any focal correction by setting the focal power of the tunable lens to zero during image acquisition. Clearly the image is not in focus everywhere; the spot sharpness changes gradually along the x-axis where only 13 spots in the middle part are in good focus and the remaining 40 spots become increasing blurred with the distance away from the region of sharp focus.

The spots acquired out of focus are significantly different from those acquired with sharp focus. It is in fact difficult to distinguish some of these blurred spots and they more or less connected with one another along the x-axis at some point. The signal amplitude of the blurred spots is significantly smaller than expected. For example, the amplitudes of the spots on the right side in Figure 2a are only 20–30% of those for the spots in the middle.

To quantify the image sharpness, we use a squared gradient function to return a value indicative of the relative sharpness of focus. It is based on the premise that well-focused images contain more information than unfocused images do [25]. The squared gradient function has the form:

(2) $$f\left(I\right)=\underset{x}{\sum }\underset{y}{\sum }\left\{{\left[I\left(x+1,\;y\right)-I(x,\;y)\right]}^{2}+{\left[I\left(x,\;y+1\right)-I(x,\;y)\right]}^{2}\right\}$$

where I(x, y) is the grey level of the image at point (x, y). From left to right along the x-axis, we divide the image in Figure 2a into 20 subunits with each unit having 35 × 1500 pixels. The squared gradient function for each subunit is shown in Figure 2b. The eighth subunit has the largest value and thus is the best focused. The values of the squared gradient functions for the subunits on the left and right are much smaller, confirming that those subunits are not well focused. Similarly, we divide the image into 20 subunits from top to bottom along the y-axis, with each unit having 710 × 75 pixels, and calculate the squared gradient function for each subunit, as shown in Figure 2c. In this case, the values of the squared gradient functions are close for all subunits, confirming that the image or spot sharpness does not change along the y-axis, as expected.

**Figure 3:**
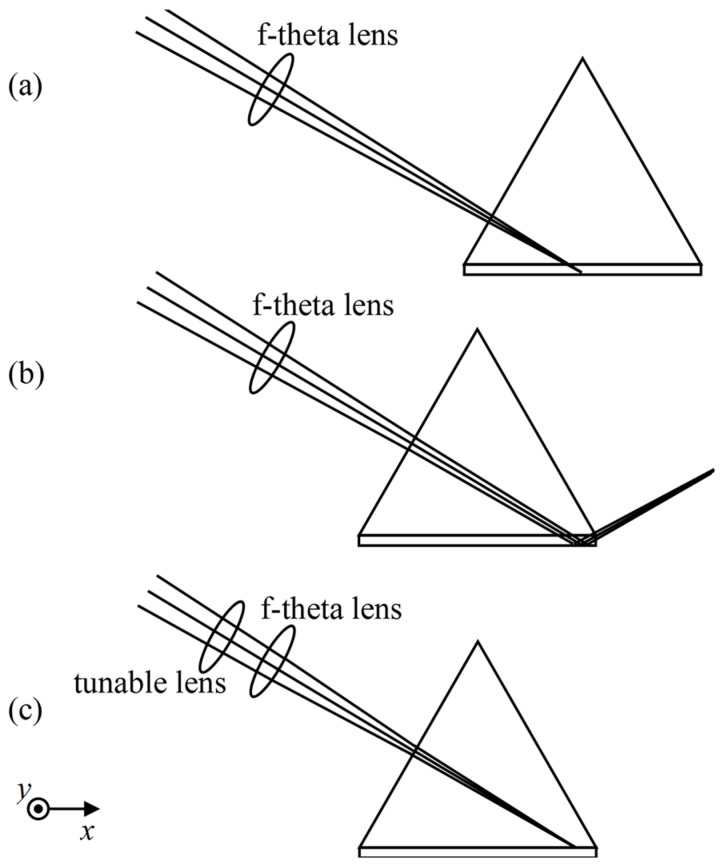
Schematic diagram showing the movement of the focal point of the illumination beam as the prism moves along the x-axis and the correction of the focal point achieved with an electronically tunable lens. (**a**) Illumination beam enters the prism and is focused on the back surface of glass slide; (**b**) As the prism moves towards the f-theta lens, the illumination beam is focused, but only after reflection from the back surface; (**c**) The tunable lens brings the focal point for the illumination beam back onto the back surface of the glass slide.

The change in spot sharpness as the translation stage moves along the *x*-axis is the result of the focal point of the illumination beam moving into and out of the microarray surface, as illustrated in Figure 3. In Figure 3a, the illumination beam enters the f-theta lens, refracting at one face of the prism, and focuses on the back surface of the glass slide bearing the microarray. As the translation stage moves along the *x*-axis to the left in Figure 3b, the illumination beam focuses after reflection from the microarray surface, making the microarray features in the image blurred.

### 3.2. Characterization of the Combination of a Tunable Lens and the Fixed F-Theta Lens

**Figure 4:**
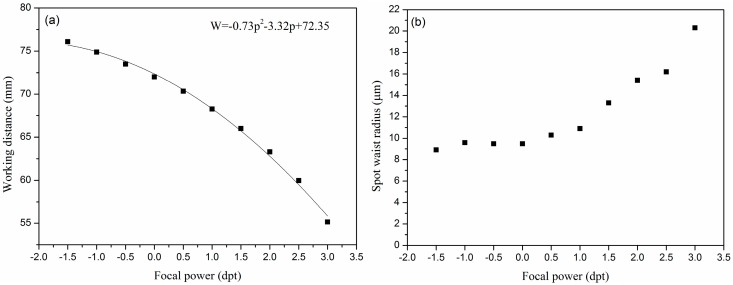
(**a**) Measured working distance as a function of focal power of the tunable lens. The zero focal power corresponds to an infinite focal length for the tunable lens. In this case, the focus of the lens combination is at the working distance of the f-theta lens alone; (**b**) Measured waist radius at the focal point of the lens combination as a function of the focal power of the tunable lens.

To keep the focal point of the illumination beam on the microarray-bearing surface (the back surface of the glass slide), we insert the electronically tunable lens 170 mm before the f-theta lens, as shown in Figure 3c. Without the tunable lens and using the scanning knife edge method, the f-theta lens is found to focus the illumination beam into a waist radius of w_0_ = 9.5 ± 0.5 μm at a working distance of 72 ± 1 mm with a Raleigh range of Z_R_ = 448 ± 50.8 μm. With the tunable lens inserted, we measure the working distance and the waist radius at the focal point of the lens combination as a function of the focal power of the tunable lens. The results are shown in Figure 4a,b, respectively.

With the focal power of the tunable lens varying from −1.5 dpt to 3.0 dpt, the working distance varies from 76 mm to 55 mm. As shown in Figure 4a, the working distance (W) as a function of the focal power (p) is fitted to a polynomial:

W = −0.73p^2^ − 3.32p + 72.35 (3)

with R^2^ = 0.996. The waist radius is more or less a constant when the focal power varies from −1.5 dpt to 1 dpt. It changes noticeably when the focal power becomes larger than 1 dpt. The increase in waist radius is due to the reduced beam diameter as it enters the f-theta lens. In addition, the vertical orientation of the tunable lens results in aberrations due to gravity on the lens. Therefore, −1.5 dpt to 1 dpt is the practical range to maintain an acceptable image quality in our current TIR OI-RD scanning microscope. This range provides us with a focus point displacement of 8 mm. The latter satisfies the need for the focal point correction when the prism is translated by 13 mm.

### 3.3. TIR OI-RD Image of the Protein Microarray with Focal Point Correction

**Figure 5:**
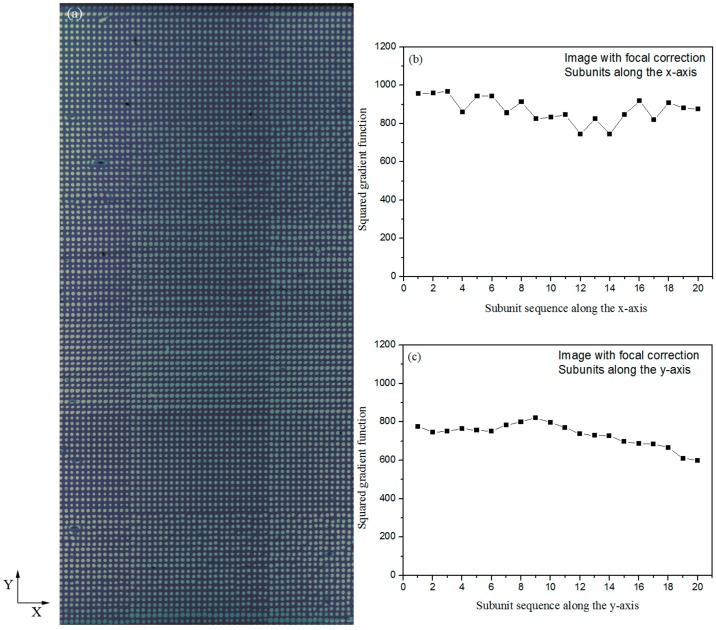
(**a**) An image of the same BBSA microarray obtained with the TIR OI-RD microscope and focal correction; (**b**) Squared gradient functions of 20 subunits along the x-axis with each unit having 35 × 1500 pixels. Subunit sequence begins from the left side of the microarray image; (**c**) Squared gradient functions of 20 subunits along the y-axis with each unit having 710 × 75 pixels. Subunit sequence begins from the top side of the microarray image.

As schematically demonstrated in Figure 3c, the focal point of the illumination beam is brought back onto the microarray surface with a tunable lens. By changing the focal power 0.03 dpt for every 280-μm movement of the translation stage, from 0.75 dpt to −0.69 dpt, we are able to obtain well-focused microarray images. In Figure 5a, we show an OI-RD image of the same protein microarray as that depicted in Figure 2a, but acquired with the focal correction. Features are sharp with comparable amplitudes throughout the whole microarray. To quantify the image sharpness from left to right along the *x*-axis, we again divide Figure 5a into 20 subunits, with each unit having 35 × 1500 pixels, and calculate squared gradient functions. The results are shown in Figure 5b. From top to bottom we also divide the image into 20 subunits, with each unit having 710 × 75 pixels, and calculate squared gradient functions. The results are displayed in Figure 5c. Small variations of the squared gradient functions along both directions confirm that all spots in Figure 5a are in focus.

### 3.4. Association-Dissociation Curves of Biochemical Reactions on a Solid Support Acquired with the Focus-Corrected TIR OI-RD Scanning Microscope

**Figure 6:**
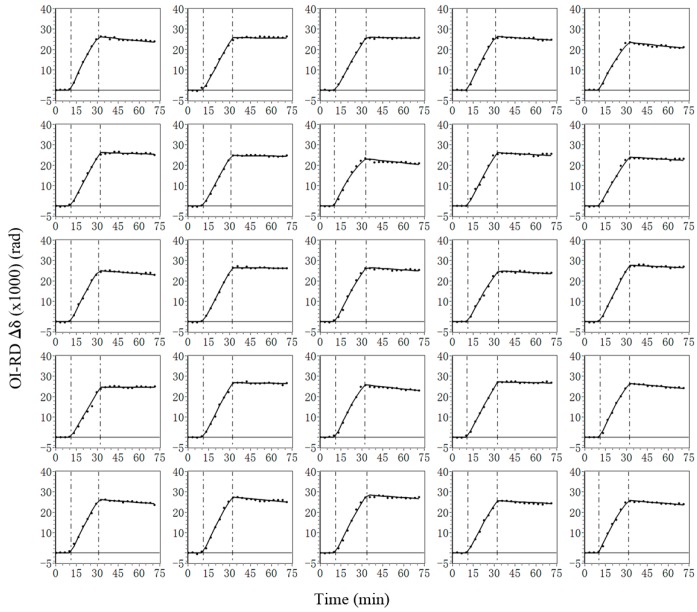
Twenty-five out of 5936 simultaneously acquired association-dissociation curves (solid circles) during incubation of the BBSA microarray in a solution of anti-biotin antibody. Vertical lines mark starts of association and dissociation phases, respectively. Solid lines through data points are fit to a 1-to-1 Langmuir reaction model.

To illustrate the stability and repeatability of the tunable lens, we incubate the BBSA microarray in a solution of anti-biotin antibody at a concentration of 62.5 nM and use the TIR OI-RD scanning microscope to acquire the association-dissociation curves on these BBSA features. During the measurement, the focal power needs to be adjusted by 0.03 dpt for every 250-μm movement of the translation stage. The adjustment is repeated for each time point of the association-dissociation curves. In the present measurement, the set of focal power values is repeated 94 times, corresponding to the number of data points in each association-dissociation curve. A subset of 5936 simultaneously acquired association-dissociation curves is shown in Figure 6. Solid lines are fit to a 1-to-1 Langmuir reaction model to yield the equilibrium dissociation constant. From the left column to the right column, the curves correspond to reactions with BBSA spots printed on the microarray from left to right, respectively. All 25 curves show similar shapes and terminal values at the end of the association, indicating that the focal correction is stable and reproducible. From these curves, we determine the equilibrium dissociation constant of anti-biotin antibody with immobilized biotinylated BSA to be 6.7 nM ± 1.2 nM.

## 4. Discussion and Conclusions

In the present work, we demonstrate that focal correction in an oblique-incidence scanning microscope can be achieved by using an electronically tunable lens or a lens combination containing such a lens. The illumination optics for such a microscope can thus be made more compact, cost-effective, and not subject to the noise and background drift associated with mechanical movement. Furthermore, the electronic tuning of focal power is much faster than mechanically moving an illumination optics. The downside of an electronically tunable lens (aberrations due to gravity with optical axis horizontal) can be removed if the lens is used with its optical axis oriented vertically, thus making the range of the focal point correction much larger than that reported in this work.
